# Efficacy and safety of live combined *Bacillus subtilis* and *Enterococcus faecium* in patients with constipation: a meta-analysis of randomized controlled trials

**DOI:** 10.3389/fphar.2025.1688544

**Published:** 2025-10-15

**Authors:** Wenwen Li, Yingying Liang, Guangcai Li, Dandan Yang, Xiaoqian Zhang

**Affiliations:** Department of Gastroenterology, Affiliated Hospital of Shandong Second Medical University, Shandong Second Medical University, Weifang, China

**Keywords:** constipation, live combined *Bacillus subtilis* and *Enterococcus faecium*, efficacy, safety, meta-analysis

## Abstract

**Background:**

Live combined *Bacillus subtilis* and *Enterococcus faecium* (LCBE) provides favorable clinical benefits in patients with constipation, although a comprehensive evaluation is lacking. This meta-analysis aimed to thoroughly evaluate the efficacy and safety of LCBE in patients with constipation.

**Methods:**

Randomized controlled trials (RCTs) assessing the efficacy or safety of LCBE in patients with constipation, published before September 2025, were comprehensively searched for in Wan Fang, China National Knowledge Infrastructure, China Science and Technology Journal Database, Web of Science, Cochrane Library, and PubMed. Efficacy and safety outcomes were extracted.

**Results:**

A total of 32 studies were included, containing 1,565 patients receiving LCBE and control treatment (experimental group) and 1,490 patients receiving control treatment alone (control group). The experimental group showed a higher total effective rate [odds ratio (OR) (95% confidence interval (CI)) = 5.789 (4.598–7.288); *p* < 0.001], Bristol Stool Scale score [standardized mean difference (SMD) (95% CI) = 2.532 (1.274–3.790); *p* < 0.001], defecation frequency per week [SMD (95% CI) = 1.937 (1.252–2.623); *p* < 0.001], and defecation rate within 24 h [OR (95% CI) = 2.545 (1.377–4.705); *p* = 0.003] than the control group. The defecation difficulty score tended to decrease in the experimental group relative to that in the control group, although this did not reach statistical significance [SMD (95% CI) = −1.924 (−3.947 to 0.099); *p* = 0.062]. There was no difference in the total adverse reaction rate between groups [OR (95% CI) = 0.703 (0.414–1.191); *p* = 0.190]. Subgroup analyses suggested that LCBE was effective, regardless of dosage form or treatment course. All studies were of moderate-to-high quality.

**Conclusion:**

LCBE demonstrates a favorable efficacy and good tolerability in patients with constipation. This meta-analysis provides supportive evidence for its clinical application in the management of constipation.

## 1 Introduction

Constipation is a disorder of the gastrointestinal system characterized by difficult and infrequent defecation, typically occurring three times or fewer per week (Bisht et al., 2023; Ihara et al., 2025; Luo et al., 2025). It affects approximately 15.0% of the global population, including both children and adults (Rao et al., 2022; Vriesman et al., 2020). Constipation usually causes ongoing physical and psychological distress to patients, which seriously affects their daily lives and quality of life (Barbara et al., 2023; Sadler et al., 2022). The common treatments for constipation include conservative therapies (basic lifestyle and dietary modifications) and pharmacological therapies (laxatives, secretagogues, and prokinetic agents) (Aziz et al., 2020; Bharucha and Lacy, 2020; Sharma and Rao, 2017).

Probiotics are beneficial microorganisms for human health and have shown clinical benefits in treating a series of gastrointestinal disorders, including constipation (Huang et al., 2025; Rau et al., 2024). Live combined *Bacillus subtilis* and *Enterococcus faecium* (LCBE), containing *B. subtilis* R-179 and *E. faecium* R-026, serves as a pioneer probiotic that helps maintain intestinal microbial balance (Huang et al., 2023; Liu et al., 2021). LCBE has two forms (granule form and capsule form): for the granule form, a packet weighs 1.0 g, including 1.5 × 10^7^ *B. subtilis* and 1.35 × 10^8^ *E. faecium*; for the capsule form, a capsule weighs 250 mg, including 5.0 × 10^7^ *B. subtilis* and 4.5 × 10^8^ *E. faecium*. At present, many studies have reported that LCBE showed a favorable efficacy with a tolerable safety profile in treating pediatric or adult patients with constipation (Cao et al., 2012; Deng and Huang, 2011; Fu and Huang, 2013; Ge et al., 2014; Guo, 2017; Huang et al., 2014; Lv et al., 2017; Wu X. L. et al., 2016; Zhang, 2019). LCBE is commercially available and has been widely applied for constipation in China, but the findings from previous LCBE-related studies are not consistent, and most studies have small sample sizes; therefore, a definitive pooled conclusion regarding its efficacy and safety is lacking.

Subsequently, this meta-analysis reviewed data from available randomized controlled trials (RCTs), aiming to comprehensively investigate the efficacy and safety of LCBE in treating patients with constipation.

## 2 Materials and methods

### 2.1 Literature strategy

A comprehensive search was conducted across multiple databases, including Wan Fang, China National Knowledge Infrastructure (CNKI), China Science and Technology Journal Database (VIP), Web of Science, Cochrane Library, and PubMed. The search keywords included “*Bacillus Subtilis* and *Enterococcus faecium*,” “Live Combined *Bacillus Subtilis* and *Enterococcus faecium*,” “Medilac-Vita,” “Medilac-S,” “constipation,” and “astriction.” Studies published before September 2025 were considered. Additionally, the references of the selected studies were manually reviewed to identify any further relevant studies. The search was performed using free-text words and combining MeSH terms in English databases. This meta-analysis was not pre-registered on an international platform. This meta-analysis adhered to the Preferred Reporting Items for Systematic Reviews and Meta-Analyses (PRISMA) reporting guidelines.

### 2.2 Literature selection criteria

Eligible studies met the following criteria: a) participants diagnosed with constipation; b) comparison of efficacy or safety between LCBE plus control treatment and control treatment alone; and c) published in English or Chinese. Studies were excluded if they met the following criteria: a) reviews, meta-analyses, or case reports; b) non-RCTs; or c) experiments (cell or animal studies).

### 2.3 Data extraction and quality assessment

Extracted data comprised the first author, publication year, sample size, demographics, intervention, dosage form of LCBE, and treatment course. The efficacy or safety outcomes of LCBE in constipation treatment were systematically screened and evaluated. Outcomes reported in fewer than three studies were excluded from the meta-analysis to ensure robustness. The quality assessment of eligible studies was assessed by two independent researchers using the ROB 2.0 tool (Sterne et al., 2019), and any inconsistencies were subsequently discussed to achieve a consensus.

### 2.4 Statistics analysis

Data analyses were performed using R software (version 4.4.2). Effect sizes were expressed as odds ratio (OR) with 95% confidence interval (CI) for categorical variables and as standardized (std.) mean differences (SMD) with 95% CIs for continuous variables. Model selection was based on the I^2^ statistic: a random-effects model was applied if I^2^ exceeded 50%, indicating significant heterogeneity; otherwise, a fixed-effects model was used. Publication bias was assessed using Peters’ and Egger’s tests for categorical and continuous variables, respectively. A *p*-value < 0.05 suggested potential publication bias, prompting adjustment using the trim-and-fill method. Sensitivity analyses were performed to assess result stability by sequentially excluding individual studies.

## 3 Results

### 3.1 Selection process

**FIGURE 1:**
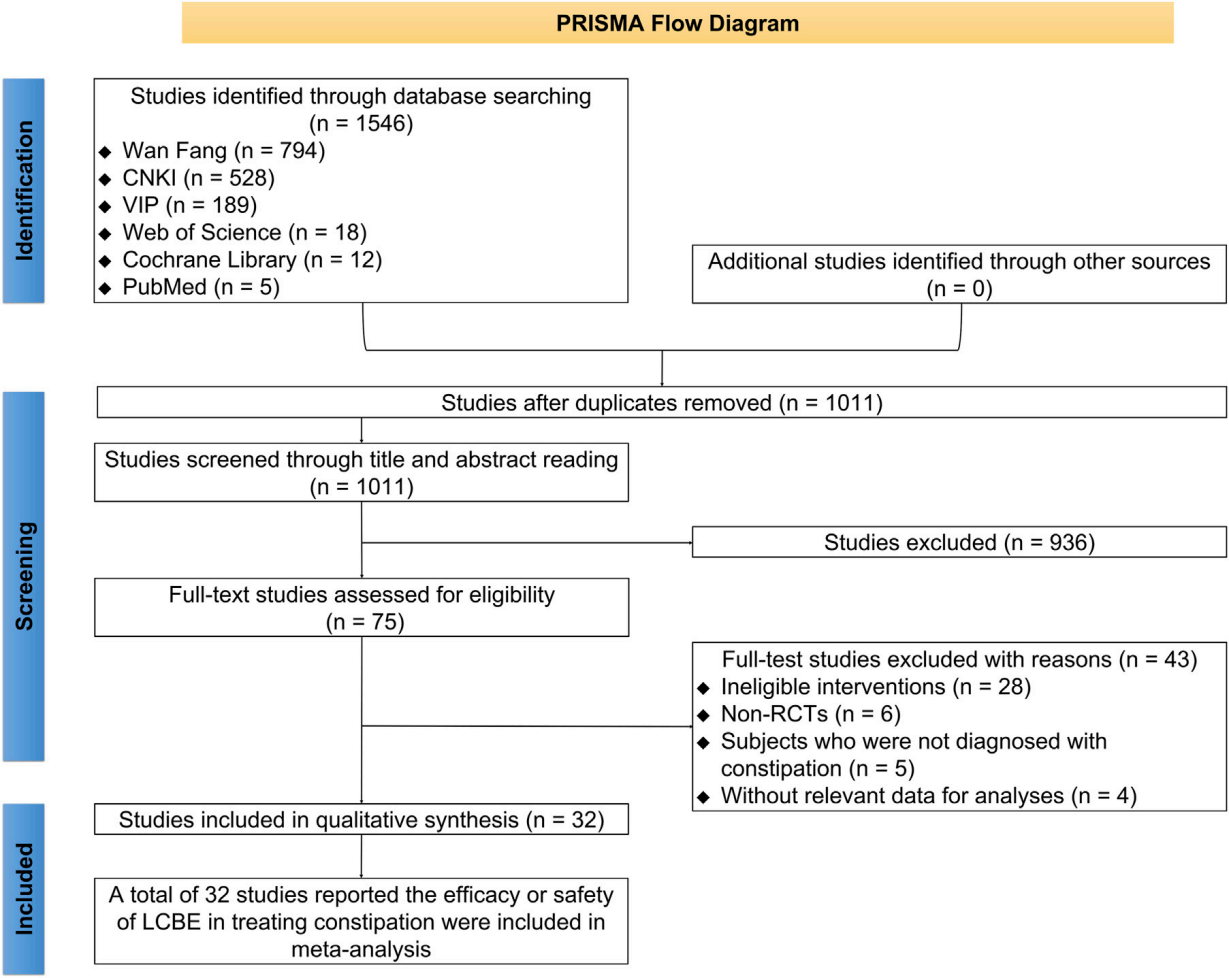
Screening flowchart.

There were 1,502 studies identified through database searching, comprising 782 studies from Wan Fang, 499 from CNKI, 189 from VIP, 16 from Web of Science, 11 from Cochrane Library, and 5 from PubMed. Subsequently, 524 duplicated studies were removed. A total of 903 studies were excluded after screening titles and abstracts. Then, 43 studies were removed after full-text reading, comprising 28 studies with ineligible interventions, 6 non-RCTs, 5 studies whose subjects were not diagnosed with constipation, and 4 studies without relevant data for analysis. Eventually, 32 studies reporting the efficacy or safety of LCBE in treating constipation were included in this meta-analysis (Cao et al., 2012; Chen and Cui, 2014; Chen et al., 2013; Cheng and Liu, 2007; Deng and Huang, 2011; Fu and Huang, 2013; Ge et al., 2014; Guo, 2017; He et al., 2020; Huang et al., 2014; Kong and Zhou, 2015; Liang et al., 2016; Liang et al., 2019; Liu et al., 2013; Lv et al., 2017; Ma et al., 2013; Mao, 2013; Peng, 2014; Peng, 2016; Qi, 2018; Tang et al., 2009; Wang, 2020; Wu et al., 2012; Wu Q. F. et al., 2016; Wu X. L. et al., 2016; Yang, 1999; Yi, 2008; Zeng and Wei, 2022; Zhang, 2019; Zhang, 2016; Zhang et al., 2017; Zhong, 2001) (Figure 1).

### 3.2 Study characteristics

The 32 studies were published between 1999 and 2022, containing 1,565 patients receiving LCBE and control treatment (experimental group) and 1,490 patients receiving control treatment alone (control group). Among these studies, LCBE was delivered in the granule form in 16 studies and the capsule form in the remaining 16 studies. The age of the participants ranged from 0.1 to 92.0 years, while the male percentage ranged from 35.4% to 65.7%; moreover, control treatments included conventional therapy, lactulose, and Liuwei-Anxiao, and the duration of LCBE treatment ranged from 2 to 8 weeks (Table 1).

**TABLE 1:** Characteristics of included studies.

Study id	Sample size		Age (years)		Male\female		Intervention		LCBE form	Treatment course
First author [publication year]	Experimental group	Control group	Experimental group	Control group	Experimental group	Control group	Experimental group	Control group		
Yang (1999)	40	40	Total: 0.1–3.0		Total: 42\38		LCBE + conventional	Conventional	Granules	2 weeks
Zhong (2001)	112	76	Total: 0.1–3.0		60\52	43\33	LCBE + conventional	Conventional	Granules	2 weeks
Cheng and Liu (2007)	52	50	Total: 0.1–3.0		Total: 67\35		LCBE + conventional	Conventional	Granules	2 weeks
Yi (2008)	44	44	Total: 0.1–14.0		Total: 52\36		LCBE + conventional	Conventional	Granules	2 weeks
Tang et al. (2009)	33	32	18.0–67.0	18.0–69.0	12\21	11\21	LCBE + Liuwei-Anxiao	Liuwei-Anxiao	Capsules	4 weeks
Deng and Huang (2011)	31	25	Total: 31.0–87.0		Total: 46\37		LCBE + lactulose	Lactulose	Capsules	4 weeks
Cao et al. (2012)	32	30	Total: 18.0–80.0		NR	NR	LCBE + lactulose	Lactulose	Capsules	2 weeks
Wu et al. (2012)	60	60	Total: 14.0–90.0		Total: 48\72		LCBE + lactulose	Lactulose	Capsules	4 weeks
Chen et al. (2013)	38	37	Mean: 4.7	Mean: 4.9	23\15	20\17	LCBE + conventional	Conventional	Granules	3 weeks
Fu and Huang (2013)	46	42	Mean: 39.5	Mean: 37.3	Total: 40\48		LCBE + lactulose	Lactulose	Capsules	4 weeks
Liu et al. (2013)	30	30	Total: 60.0–75.0		Total: 42\48		LCBE + lactulose	Lactulose	Capsules	4 weeks
Ma et al. (2013)	119	107	Total: 38.0–85.0		Total: 108\118		LCBE + macrogol 4000 powder	Macrogol 4000 powder	Capsules	6 weeks
Mao (2013)	40	40	Total: 0.3–4.0		26\14	24\16	LCBE + conventional	Conventional	Granules	2 weeks
Chen and Cui (2014)	60	60	3.0–12.0	2.0–11.0	32\28	29\31	LCBE + lactulose	Lactulose	Granules	NR
Ge et al. (2014)	46	30	Total: 65.0–92.0		Total: 66\42		LCBE + lactulose	Lactulose	Capsules	4 weeks
Huang et al. (2014)	31	28	18.0–74.0	20.0–76.0	15\16	14\14	LCBE + Liuwei-Anxiao	Liuwei-Anxiao	Capsules	4 weeks
Peng (2014)	46	46	Mean: 7.3	Mean: 7.6	25\21	24\22	LCBE + conventional	Conventional	Granules	4 weeks
Kong and Zhou (2015)	28	27	25.0–78.0	27.0–80.0	17\11	15\12	LCBE + lactulose	Lactulose	Capsules	4 weeks
Liang et al. (2016)	35	35	Mean: 68.3	Mean: 68.1	12\23	13\22	LCBE + lactulose	Lactulose	Capsules	4 weeks
Peng (2016)	28	24	0.1–3.0	0.1–3.0	NR	NR	LCBE + lactulose	Lactulose	Granules	2 weeks
Wu et al. (2016a)	60	60	Mean: 2.9	Mean: 3.1	34\26	30\30	LCBE + lactulose	Lactulose	Granules	8 weeks
Wu et al. (2016b)	56	56	24.0–81.0	26.0–85.0	32\24	35\21	LCBE + lactulose	Lactulose	Capsules	4 weeks
Zhang (2016)	29	29	2.0–8.0	1.0–8.0	12\17	11\18	LCBE + conventional	Conventional	Granules	2 weeks
Guo (2017)	50	50	>18.0	>18.0	NR	NR	LCBE + Testa triticum tricum purif	Testa triticum tricum purif	Capsules	20 days
Lv et al. (2017)	83	97	NR	NR	NR	NR	LCBE + mosapride	Mosapride	Capsules	4 weeks
Zhang et al. (2017)	56	56	Total: mean: 7.5		Total: 74\38		LCBE + conventional	Conventional	Granules	2 weeks
Qi (2018)	50	50	60.0–79.0	60.0–78.0	23\27	22\28	LCBE + lactitol	Lactitol	Capsules	4 weeks
Liang et al. (2019)	44	44	Mean: 2.4	Mean: 2.4	28\16	26\18	LCBE + lactulose	Lactulose	Granules	8 weeks
Zhang (2019)	44	43	61.0–86.0	60.0–85.0	25\19	23\20	LCBE + lactulose	Lactulose	Capsules	4 weeks
He et al. (2020)	52	52	4.0–9.0	4.0–9.0	29\23	27\25	LCBE + lactulose	Lactulose	Granules	3 weeks
Wang (2020)	30	30	1.0–6.0	1.0–7.0	16\14	18\12	LCBE + lactulose	Lactulose	Granules	NR
Zeng and Wei (2022)	60	60	0.5–7.0	0.5–8.0	29\31	27\33	LCBE + conventional	Conventional	Granules	2 weeks

LCBE, live combined *Bacillus subtilis* and *Enterococcus* faecium; NR, not reported.

Special statement: 1) Age was described as the range or mean value. 2) The “Total” in parentheses indicated that the data described the overall study population, and there was no separate report of experimental and control groups in the original study. 3) The “Conventional” indicated conventional treatment reported in the original studies, which involved more hydration, dietary modifications, bowel habit training, and so on.

### 3.3 Quality assessment

**FIGURE 2:**
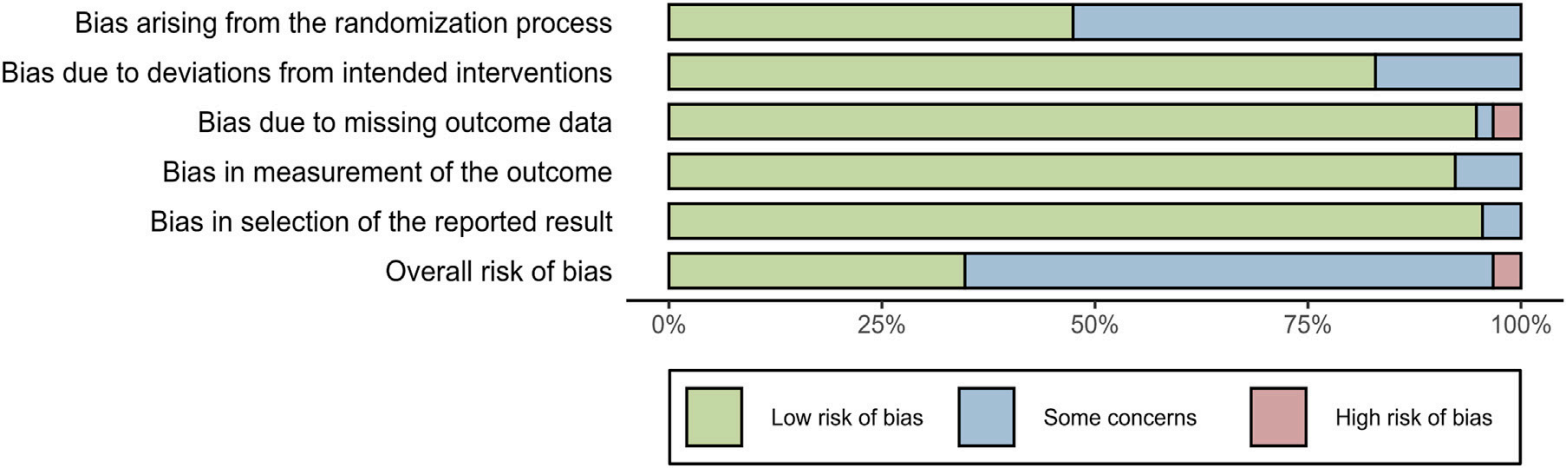
Assessment of the risk of bias.

The quality of included studies was evaluated using the ROB 2.0 tool. For bias arising from the randomization process, 16 studies were assessed as “low risk of bias,” and the remaining 16 studies were evaluated as having “some concerns.” Regarding bias due to deviations from intended interventions, 26 studies were assessed as “low risk of bias,” and 6 studies were evaluated as having “some concerns.” For bias due to missing outcome data, 30 studies were assessed as “low risk of bias,” 1 study was evaluated as having “some concerns,” and 1 study was evaluated as “high risk of bias.” For bias in the measurement of the outcome, 29 studies were assessed as “low risk of bias,” and 3 studies were evaluated as having “some concerns.” For bias in the selection of the reported results, 30 studies were evaluated as “low risk of bias,” and 2 studies were evaluated as having “some concerns.” In terms of overall risk of bias, there were 10 studies assessed as “low risk of bias,” 21 studies evaluated as having “some concerns,” and 1 study categorized into “high risk of bias.” The above information indicated that the included studies were of moderate-to-high quality (Figure 2).

### 3.4 Total effective rate

**FIGURE 3:**
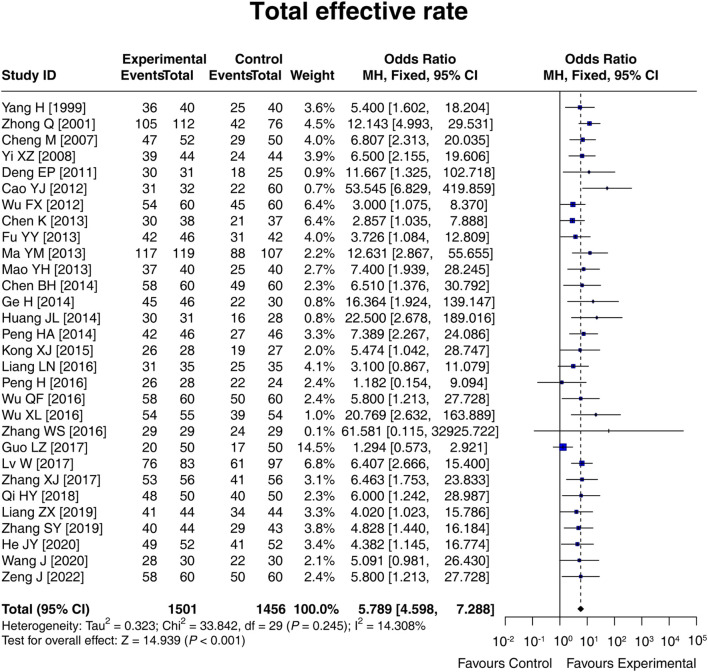
Forest plot of the total effective rate between groups.

A total of 30 studies compared the total effective rate between the experimental and control groups, and there was no heterogeneity among these studies (I^2^ = 14.308%; *p* = 0.245). The fixed-effects model showed that the total effective rate in the experimental group was 5.789 times that in the control group [OR (95% CI): 5.789 (4.598, 7.288); *p* < 0.001] (Figure 3). Moreover, subgroup analysis based on the control intervention revealed that the total effective rate was higher in the experimental group than in the control group, regardless of whether the control intervention was conventional therapy [OR (95% CI): 6.774 (4.646, 9.876); *p* < 0.001], lactulose [OR (95% CI): 5.766 (3.983, 8.347); *p* < 0.001], or others [OR (95% CI): 4.563 (2.848, 7.311); *p* < 0.001] (Supplementary Figure S1). The result disclosed that LCBE elevated the total effective rate.

### 3.5 Indices related to treatment efficacy

**FIGURE 4:**
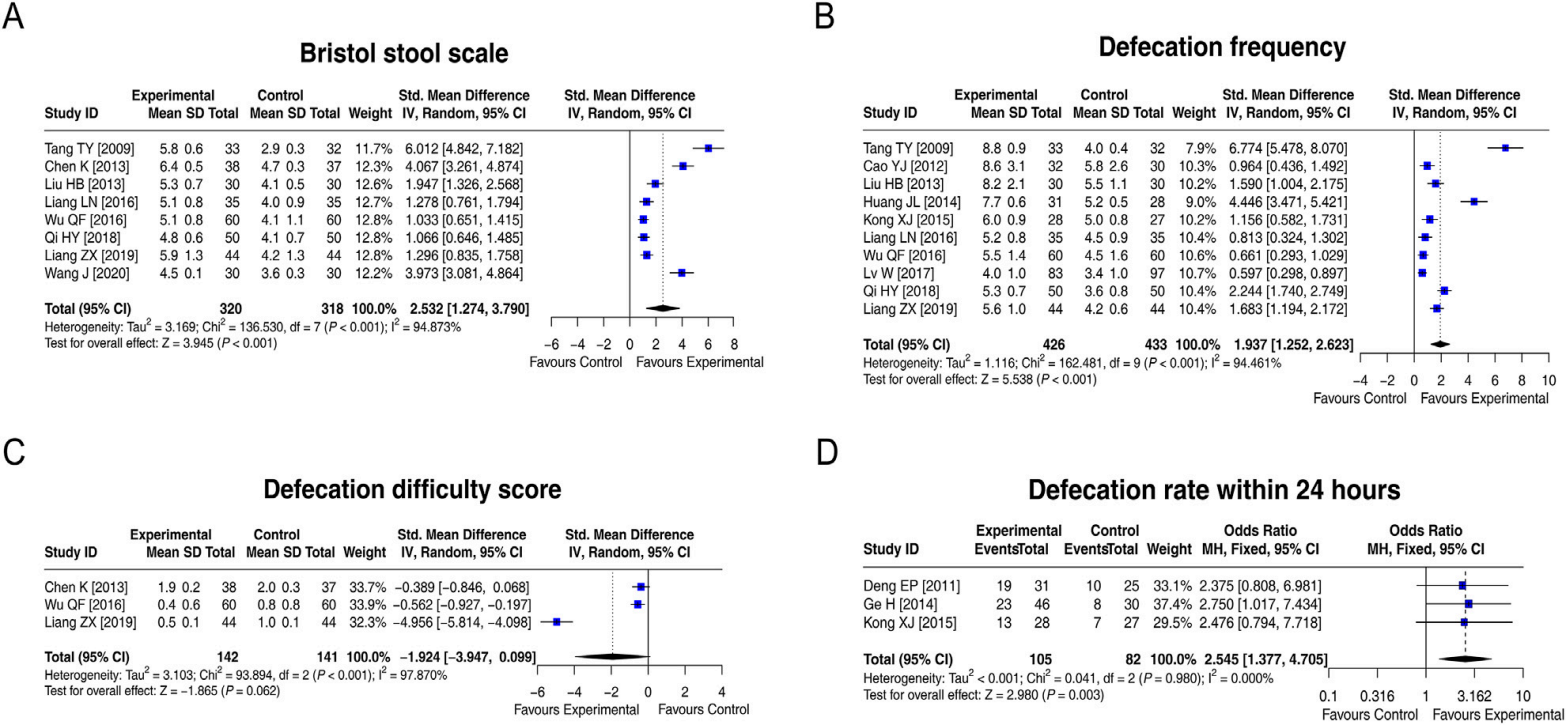
Forest plots of indices related to treatment efficacy between groups. Forest plots of the Bristol Stool Scale score **(A)**, defecation frequency per week **(B)**, defecation difficulty score **(C)**, and defecation rate within 24 h **(D)** between groups.

Eight studies compared the Bristol Stool Scale score between groups, and heterogeneity existed among these studies (I^2^ = 94.873%; *p* < 0.001). The random-effects model found that the Bristol Stool Scale score in the experimental group was increased by 2.532 standard deviation units compared to that in the control group [SMD (95% CI): 2.532 (1.274, 3.790); *p* < 0.001] (Figure 4A). Ten studies compared the defecation frequency per week between groups. Heterogeneity was found among these studies (I^2^ = 94.461%; *p* < 0.001). The random-effects model indicated that the defecation frequency per week in the experimental group was higher by 1.937 standard deviation units compared to that in the control group [SMD (95% CI): 1.937 (1.252, 2.623); *p* < 0.001] (Figure 4B). Three studies compared the defecation difficulty score between groups, and heterogeneity was found among them (I^2^ = 97.870%; *p* < 0.001). The random-effects model suggested that the defecation difficulty score tended to be reduced by 1.924 standard deviation units in the experimental group compared to that in the control group, although there was no statistical significance [SMD (95% CI): −1.924 (−3.947, 0.099); *p* = 0.062] (Figure 4C). Moreover, three studies compared the defecation rate within 24 h, and no heterogeneity was observed (I^2^ = 0.000%; *p* = 0.980). The fixed-effects model showed that the defecation rate within 24 h in the experimental group was 2.545 times that of the control group [OR (95% CI): 2.545 (1.377, 4.705); *p* = 0.003] (Figure 4D). Overall, LCBE increased the Bristol Stool Scale score, defecation frequency per week, and the defecation rate within 24 h and tended to decrease the defecation difficulty score.

### 3.6 Adverse reaction rate

**FIGURE 5:**
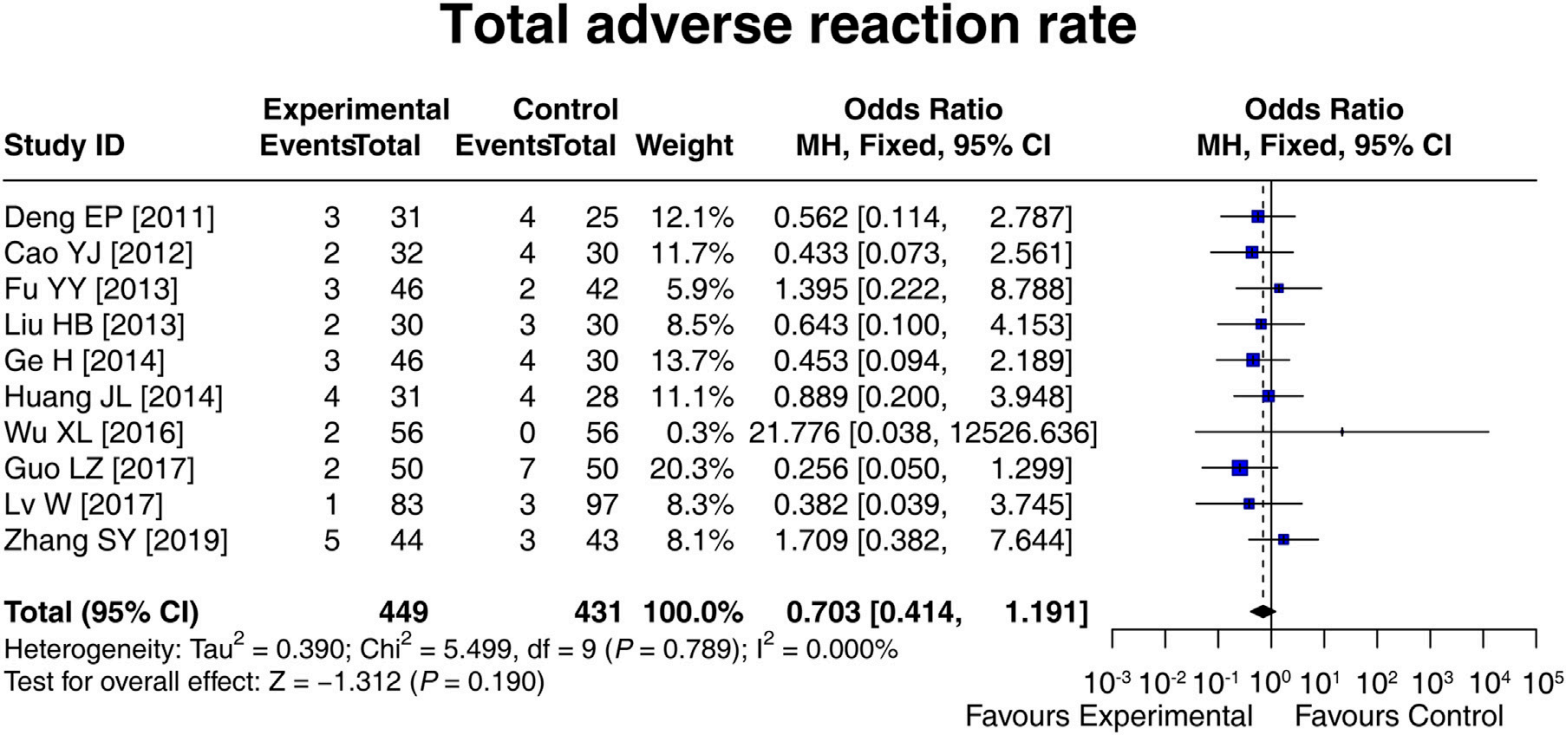
Forest plot of the total adverse reaction rate between groups.

Ten studies compared the total adverse reaction rate between the experimental and control groups, and there was no heterogeneity among these studies (I^2^ = 0.000%; *p* = 0.789). Then, the fixed-effects model showed that there was no difference in the total adverse reaction rate between groups [OR (95% CI): 0.703 (0.414, 1.191); *p* = 0.190] (Figure 5). In addition, the specific adverse reaction, including diarrhea [OR (95% CI): 0.812 (0.356, 1.852); *p* = 0.620], nausea and vomiting [OR (95% CI): 0.971 (0.243, 3.891); *p* = 0.967], abdominal distension [OR (95% CI): 0.565 (0.199, 1.610); *p* = 0.286], and abdominal pain [OR (95% CI): 1.513 (0.257, 8.901); *p* = 0.647] were not different between the experimental and control groups (Supplementary Table S1). These results suggested that LCBE did not increase the risk of adverse reactions.

### 3.7 Subgroup analysis on the total effective rate based on the dosage form of LCBE

**FIGURE 6:**
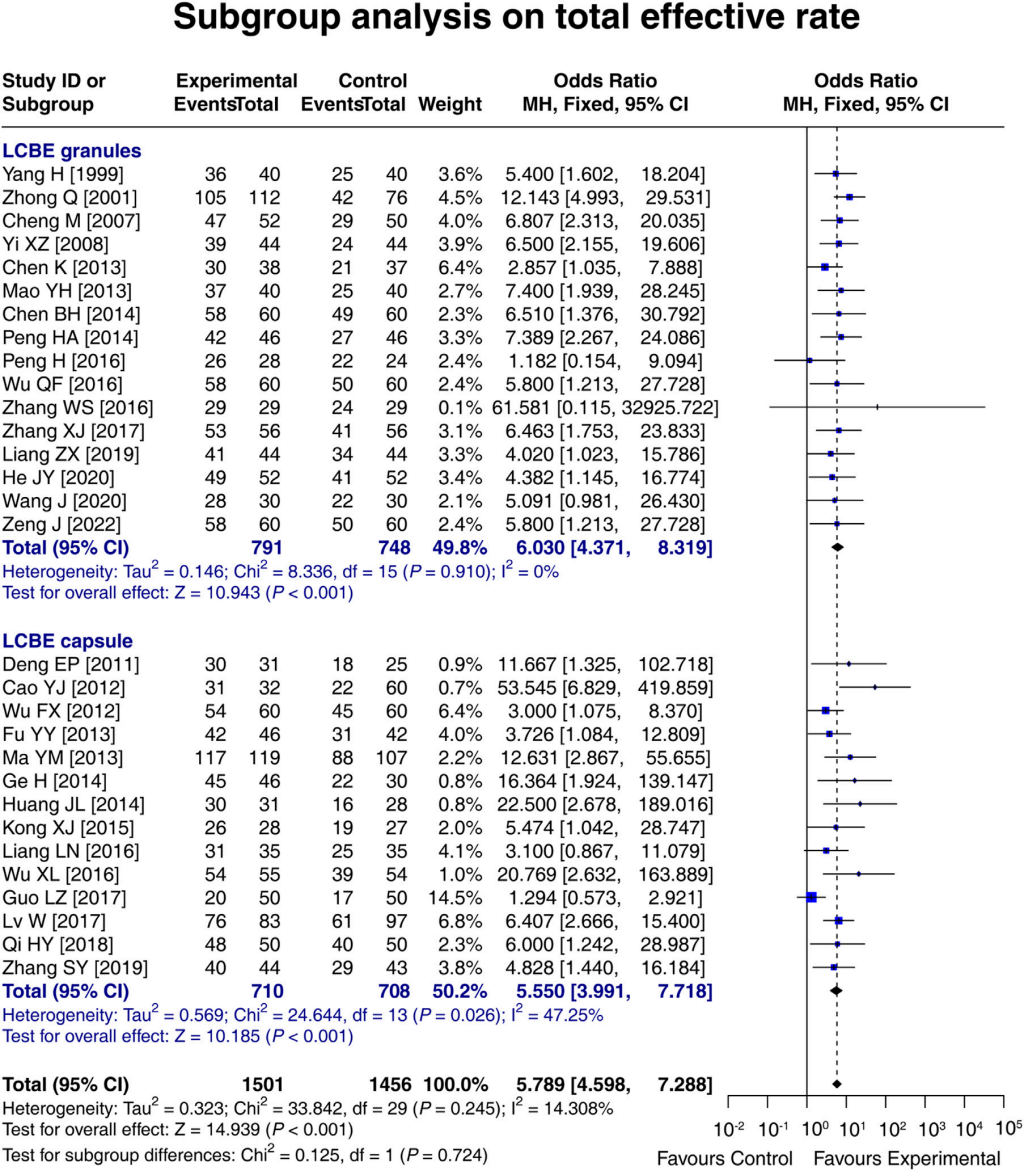
Forest plot of subgroup analysis on the total effective rate based on the dosage form of LCBE.

In 16 studies in which LCBE was administered in the granule form in the experimental group, the total effective rate in the experimental group was 6.030 times that in the control group [OR (95% CI): 6.030 (4.371, 8.319); *p* < 0.001]. In 14 studies in which LCBE was administered in the capsule form in the experimental group, the total effective rate in the experimental group was 5.550 times that in the control group [OR (95% CI): 5.550 (3.991, 7.718); *p* < 0.001] (Figure 6). Overall, both LCBE granules and capsules increased the total effective rate.

### 3.8 Subgroup analysis on the total effective rate based on the treatment course

**FIGURE 7:**
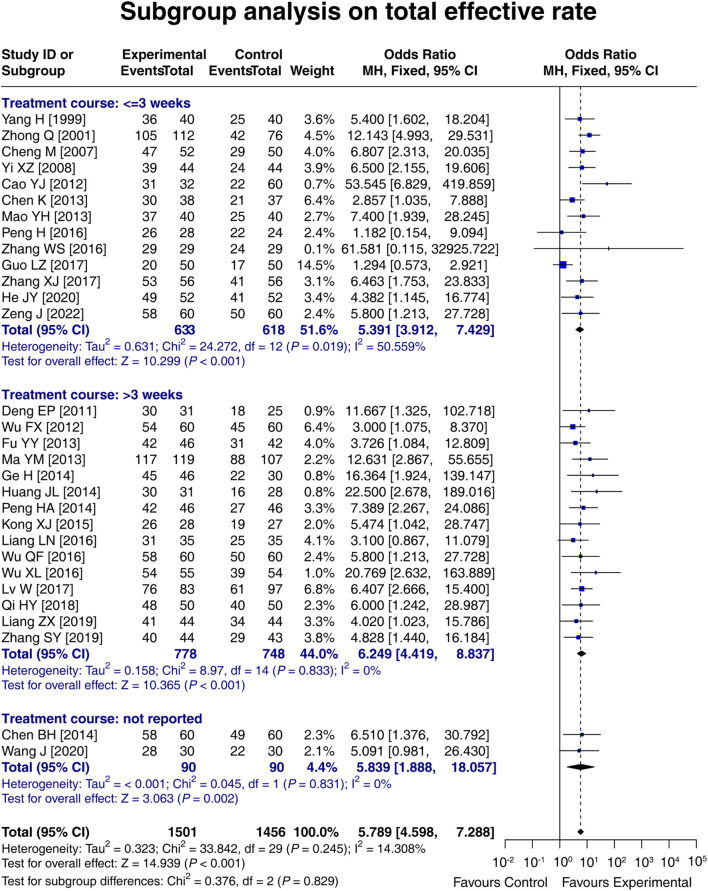
Forest plot of subgroup analysis on the total effective rate based on treatment course.

In 13 studies with a treatment course ≤3 weeks, the total effective rate in the experimental group was 5.391 times that in the control group [OR (95% CI): 5.391 (3.912, 7.429); *p* < 0.001]. In 15 studies with a treatment course >3 weeks, the total effective rate in the experimental group was 6.249 times that in the control group [OR (95% CI): 6.249 (4.419, 8.837); *p* < 0.001]. In two studies that did not report the treatment course, the total effective rate in the experimental group was 5.839 times that in the control group [OR (95% CI): 5.839 (1.888, 18.057); *p* = 0.002]. These results indicated that LCBE increased the total effective rate, regardless of the treatment course. Meanwhile, a treatment course of LCBE >3 weeks tended to increase the total effective rate compared to that of ≤3 weeks, although it did not reach statistical significance (*p* = 0.829) (Figure 7).

### 3.9 Sensitivity analyses

**FIGURE 8:**
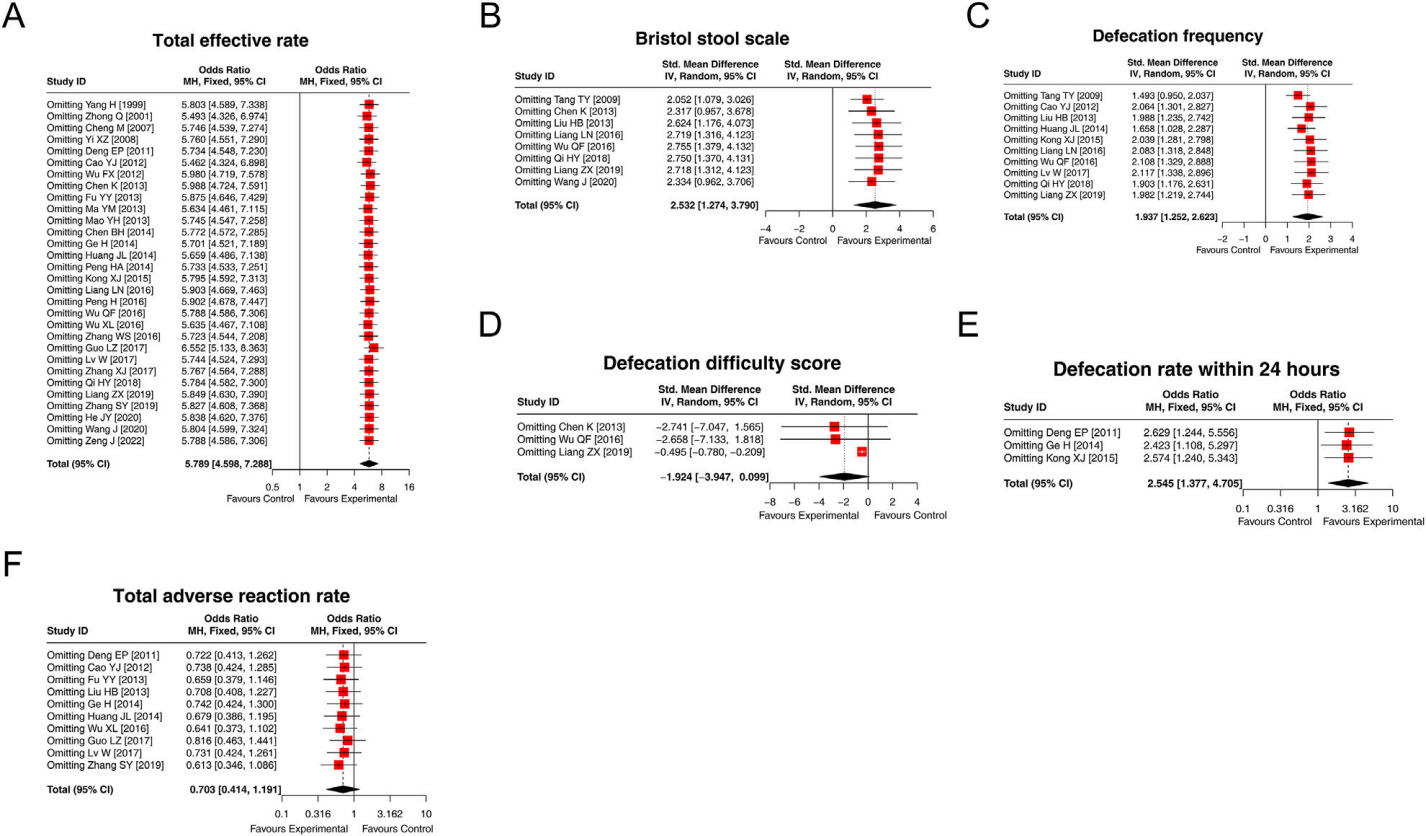
Forest plots of sensitivity analysis. The results of the total effective rate **(A)**, the Bristol Stool Scale score **(B)**, defecation frequency per week **(C)**, defecation difficulty score **(D)**, defecation rate within 24 h **(E)**, and the total adverse reaction rate **(F)** after omitting each study.

The results of the total effective rate, the Bristol Stool Scale score, the defecation frequency per week, the defecation rate within 24 h, and the total adverse reaction rate would not be affected by omitting any single study. However, excluding the study by Liang et al. (2019) would affect the result of the defecation difficulty score. Overall, the sensitivity analyses indicated high robustness of the results (Figures 8A–F).

### 3.10 Publication bias

For categorical variables, Peters’ test showed that there was no publication bias in the total effective rate (*p* = 0.730), the defecation rate within 24 h (*p* = 0.194), or the total adverse reaction rate (*p* = 0.874). In terms of continuous variables, Egger’s tests suggested that no publication bias was observed in the defecation difficulty score (*p* = 0.230). However, there were publication biases in the Bristol Stool Scale score (*p* = 0.001) and defecation frequency per week (*p* = 0.001). These publication biases were further adjusted using the trim-and-fill method. For the publication bias in the Bristol Stool Scale score, the adjusted result was different from the unadjusted result, showing that there was no significant difference between groups [SMD (95% CI): 1.61 (−0.02, 3.23)]. Regarding the publication bias in the defecation frequency per week, the adjusted results remained unchanged, revealing a difference between groups [SMD (95% CI): 1.00 (0.21, 1.79)] (Table 2).

**TABLE 2:** Publication bias.

Indicator	*p*-value	Bias estimate (SE)	Adjusted pooled estimate [95% CI]
Total effective rate	0.730 (Peters’ test)	−15.64 (44.79)	
Bristol stool scale	0.001 (Eggers’ test)	12.43 (1.17)	1.61 [−0.02, 3.23]
Defecation frequency	0.001 (Eggers’ test)	10.54 (1.90)	1.00 [0.21, 1.79]
Defecation difficulty	0.230 (Eggers’ test)	−17.12 (6.48)	
Defecation rate within 24 h	0.194 (Peters’ test)	−25.68 (8.07)	
Total adverse reaction rate	0.874 (Peters’ test)	−14.38 (87.57)	

SE, standard error; CI, confidence interval.

## 4 Discussion

Mounting evidence suggests that the occurrence of constipation is closely associated with intestinal microbiota disorder (Feng et al., 2024; Xu et al., 2024). Probiotics are live microorganisms that work to regulate the balance of intestinal microbiota (Chandrasekaran et al., 2024). Previous meta-analyses of RCTs have reported that probiotics show favorable efficacy in patients with constipation, but these meta-analyses focused on multiple probiotic strains rather than a specific probiotic strain (Deng et al., 2024; Deng et al., 2023; Dimidi et al., 2014; Ding et al., 2024; Huang and Hu, 2017; Zhang et al., 2020). As a pioneer probiotic, LCBE has gradually attracted attention for the treatment of constipation in recent years (Cao et al., 2012; Chen and Cui, 2014; Chen et al., 2013; Cheng and Liu, 2007; Deng and Huang, 2011; Fu and Huang, 2013; Ge et al., 2014; Guo, 2017; He et al., 2020; Huang et al., 2014; Kong and Zhou, 2015; Liang et al., 2016; Liang et al., 2019; Liu et al., 2013; Liu et al., 2021; Lv et al., 2017; Ma et al., 2013; Mao, 2013; Peng, 2014; Peng, 2016; Qi, 2018; Tang et al., 2009; Wang, 2020; Wu et al., 2012; Wu Q. F. et al., 2016; Wu X. L. et al., 2016; Yang, 1999; Yi, 2008; Zeng and Wei, 2022; Zhang, 2019; Zhang, 2016; Zhang et al., 2017; Zhong, 2001). This meta-analysis focused on LCBE and comprehensively assessed its efficacy in treating patients with constipation. The findings of this meta-analysis were consistent with previous studies (Deng et al., 2024; Deng et al., 2023; Dimidi et al., 2014; Ding et al., 2024; Huang and Hu, 2017; Zhang et al., 2020), revealing that LCBE increased the total effective rate. Moreover, LCBE increased the Bristol Stool Scale score, defecation frequency per week, and the defecation rate within 24 h and tended to reduce the defecation difficulty score. Possible reasons are as follows: (1) LCBE replenishes normal flora and inhibits pathogenic bacteria in the gut, which restores the intestinal homeostasis and effectively alleviates constipation (Qi, 2018; Zhang, 2019). (2) LCBE promotes lactate production and decreases the pH value in the gut, which stimulates intestinal peristalsis and facilitates the elimination of feces (Liang et al., 2019; Wang, 2020). Overall, these results indicate that LCBE is an effective probiotic for the treatment of constipation. Moreover, the improvement of the effective rate, the Bristol tool scale, and defecation frequency by LCBE would further improve the quality of life of the patients and elevate their satisfaction. However, related data could not be pooled for analysis due to the limited data available from the included studies in this meta-analysis.

Furthermore, this meta-analysis identified high levels of heterogeneity regarding “Bristol stool scale, defecation frequency, and defecation difficulty score;” the possible reasons are as follows: (1) differences in ages, ranging from 0.1 to 92.0 years; (2) differences in control treatments (conventional treatment, lactulose, Liuwei-Anxiao, etc.); (3) differences in treatment courses, ranging from 2 to 8 weeks; and (4) differences in the definitions of constipation. Moreover, there were excessive overlaps in the 95% CIs regarding the outcomes between these two studies (Wu X. L. et al., 2016; Zhang, 2016); the phenomenon might be explained as follows: sparse events would cause extremely wide CIs. In the study by Zhang WS, all 29 (100%) patients in the experimental group achieved total efficacy, with none (0%) lacking total efficacy (sparse event); in the study by Wu XL, no patients (0%) in the control group experienced adverse reactions (sparse event).

In addition to treatment efficacy, the safety assessment of LCBE in treating patients with constipation is also necessary. This meta-analysis revealed that LCBE did not elevate the total adverse reaction rate. Moreover, the included studies of this meta-analysis showed that the common adverse reactions of LCBE in patients with constipation included abdominal pain, bloating, and diarrhea, and these adverse reactions were generally mild and could be spontaneously resolved (Cao et al., 2012; Deng and Huang, 2011; Fu and Huang, 2013; Ge et al., 2014; Guo, 2017; Huang et al., 2014; Lv et al., 2017; Wu X. L. et al., 2016; Zhang, 2019). In addition, this meta-analysis also revealed that LCBE did not increase the specific adverse reactions, including diarrhea, nausea and vomiting, abdominal distension, and abdominal pain. Overall, the above results revealed a good safety profile of LCBE in patients with constipation.

LCBE can be given in two dosage forms: the granule form of LCBE is applied for pediatric patients, while the capsule form is commonly used for adult administration (Slavkova and Breitkreutz, 2015). This meta-analysis performed a subgroup analysis on the total effective rate based on the dosage form of LCBE, and the results showed that both LCBE granules and capsules could increase the total effective rate. This finding indicated that LCBE had a favorable efficacy in both pediatric and adult patients with constipation. Meanwhile, subgroup analysis also suggested that LCBE increased the total effective rate, regardless of the treatment course, and a treatment course of LCBE >3 weeks tended to increase the total effective rate compared to that of ≤3 weeks. This result indicated that both the short- and long-term efficacies of LCBE in treating constipation are outstanding.

LCBE is widely applied in China; however, studies from other regions are limited, with only a few reported in Korea (Kim et al., 2006; Lim et al., 2023). One study investigated the application of LCBE for the treatment of *Helicobacter pylori* infection in Korea and reported that LCBE did not increase the eradication rate [A]; another study investigated the use of LCBE for the treatment of irritable bowel syndrome in Korea and revealed that LCBE improved the severity and frequency of abdominal pain [B]. However, no related studies on LCBE use for constipation have been conducted in other regions apart from China.

Quality assessment for overall risk of bias revealed that 11 studies were assessed as “low risk,” 21 studies were evaluated as having “some concerns,” and no study was assessed as “high risk of bias,” indicating that all included studies were of moderate-to-high quality. There was no publication bias in most results, except for the Bristol Stool Scale score and defecation frequency per week. For the publication bias in the Bristol Stool Scale score, after adjusting for bias using the trim-and-fill method, the result was no longer significant. This finding indicated that the effect of LCBE on increasing the Bristol Stool Scale score might not be robust and requires further validation. Regarding the publication bias in the defecation frequency per week, the adjusted result remained unchanged, indicating that the result was reliable. Meanwhile, sensitivity analyses revealed a high robustness of the results.

There were some limitations in this meta-analysis: (1) there were differences in the treatment efficacy criteria for constipation among included studies, which might affect the reliability of results to some extent; (2) None of the studies included in this meta-analysis described the use of the blind method, which might cause some bias in the results; (3) Some results might be partly published in the included studies, which would affect the reliability of this meta-analysis; (4) All included studies were published in China and, due to differences in genetic backgrounds, dietary habits, and living environments, the generalizability of the findings to populations in other countries and regions may be limited. Therefore, high-quality RCTs in diverse populations are warranted for further validation in the future; and (5) This meta-analysis was not pre-registered on an international platform.

## 5 Conclusion

In conclusion, LCBE is effective and safe for patients with constipation. This meta-analysis supports the clinical use of LCBE as a promising probiotic for the management of constipation.

## Data Availability

The original contributions presented in the study are included in the article/Supplementary Material; further inquiries can be directed to the corresponding authors.

## Supplementary material

SUPPLEMENTARY FIGURE S1 Forest plot of subgroup analysis on the total effective rate based on control intervention.
