# Supplementary material for: Efficacy and safety of live combined Bacillus subtilis and Enterococcus faecium in patients with constipation: a meta-analysis of randomized controlled trials
Source: Front Pharmacol. 2025 Oct 15;16:1688544. doi: 10.3389/fphar.2025.1688544 (PMC12569391; doi:10.3389/fphar.2025.1688544)
Supplement: Supplementary file 1 [file Table1.docx]

**Supplementary Table 1.** Summaries of adverse events.

| Study ID: | Groups | | | Total adverse reaction | | Diarrhea | | Nausea and vomiting | | Abdominal distension | | Abdominal pain | |
| --- | --- | --- | --- | --- | --- | --- | --- | --- | --- | --- | --- | --- | --- |
| First author [publication year] |  |  |  | Experimental group | Control group | Experimental group | Control group | Experimental group | Control group | Experimental group | Control group | Experimental group | Control group |
| Deng EP [2011](Deng and Huang, 2011) | NR | | | 3 | 4 | (-) | (-) | (-) | (-) | (-) | (-) | (-) | (-) |
| Cao YJ [2012](Cao et al., 2012) | NR | | | 2 | 4 | (-) | (-) | (-) | (-) | (-) | (-) | (-) | (-) |
| Fu YY [2013](Fu and Huang, 2013) | NR | | | 3 | 2 | 3 | 2 | (-) | (-) | (-) | (-) | (-) | (-) |
| Liu HB [2013](Liu et al., 2013) | Elderly | | | 2 | 3 | (-) | (-) | (-) | (-) | (-) | (-) | (-) | (-) |
| Ge H [2014](Ge et al., 2014) | Elderly | | | 3 | 4 | (-) | (-) | (-) | (-) | (-) | (-) | (-) | (-) |
| Huang JL [2014](Huang et al., 2014) | NR | | | 4 | 4 | 2 | 2 | 1 | 1 | (-) | (-) | 1 | 1 |
| Kong XJ [2015](Kong and Zhou, 2015) | Adults | | | (-) | (-) | 3 | 3 | (-) | (-) | 2 | 4 | (-) | (-) |
| Liang LN [2016](Liang et al., 2016) | Elderly | | | (-) | (-) | 1 | 2 | 1 | 1 | (-) | (-) | (-) | (-) |
| Wu XL [2016](Wu et al., 2016) | NR | | | 2 | 0 | (-) | (-) | (-) | (-) | (-) | (-) | (-) | (-) |
| Guo LZ [2017](Guo, 2017) | Adults | | | 2 | 7 | 1 | 2 | (-) | (-) | 1 | 4 | (-) | (-) |
| Lv W [2017](Lv et al., 2017) | NR | | | 1 | 3 | 0 | 2 | (-) | (-) | (-) | (-) | 1 | 1 |
| Qi HY [2018](Qi, 2018) | Elderly | | | (-) | (-) | 1 | 0 | 1 | 2 | (-) | (-) | (-) | (-) |
| Zhang SY [2019](Zhang, 2019) | Elderly | | | 5 | 3 | (-) | (-) | 1 | 0 | 3 | 2 | 1 | 0 |
| **Qualitative synthesis** | | |  |  |  |  |  |  |  |  |  |  |  |
| No. of included studies | | |  | 10 | | 7 | | 4 | | 3 | | 3 | |
| Model |  | | | Fixed effect model | | Fixed effect model | | Fixed effect model | | Fixed effect model | | Fixed effect model | |
| OR [95% CI] |  | | | 0.703 [0.414, 1.191] | | 0.812 [0.356, 1.852] | | 0.971 [0.243, 3.891] | | 0.565 [0.199, 1.610] | | 1.513 [0.257, 8.901] | |
| Test for overall effect | |  | | Z = -1.312; *P* = 0.190 | | Z = -0.496; *P* = 0.620 | | Z = -0.041; *P* = 0.967 | | Z = -1.068; *P* = 0.286 | | Z = 0.458; *P* = 0.647 | |

NR, not report. (-) indicated no related data could be retrieved from the study.

**References**

Cao, Y. J., Qu, C. M., Liang, S. W., Zhong, C. Q., Li, L. Y., Wang, X. Y., et al. (2012). Clinical efficacy of lactulose combined with medilac-s in the treatment of functional constipation. *Chinese Journal of Microecology* 24, 625-627. doi: 10.13381/j.cnki.cjm.2012.07.022

Deng, E. P. & Huang, H. G. (2011). The therapeutic effects of Medilac-S combined with lactulose on chronic functional constipation. *Modern Hospital* 11, 12-13. doi: 10.3969 /j.issn.1671-332X.2011.06.005

Fu, Y. Y. & Huang, Y. Q. (2013). The therapeutic effect of living Bacillus subtilis and Enterococcus faecium combined with lactulose in treatment of chronic functional constipation. *Journal of Clinical and Experimental Medicine* 12, 507-508. doi: 10.3969/j.issn.1671-4695.2013.07.011

Ge, H., Pan, J. D., Wu, R. F., Hu, W. J. & Zang, Y. H. (2014). Therapeutic effects of Medilac-S combined with Lactulose on chronic functional constipation. *Journal of Taishan Medical College* 35, 878-879. doi: 10.3969/J.issn.1004-7115.2014.07.014

Guo, L. Z. (2017). Efficacy of Testa Triticum Tricum Purif combined with Medilac-S in the Treatment of Adult Constipation. *China Foreign Medical Treatment* 36, 128-129, 132. doi: 10.16662/j.cnki.1674-0742.2017.26.128

Huang, J. L., Rong, H. Y., Zhu, Y. L. & Zhang, M. (2014). The Therapeutic Effects of Medilac-SIVith Liuwei-Anxiao on Chronic Functional Constipation. *Guide of China Medicine* 12, 14-15. doi: CNKI:SUN:YYXK.0.2014-13-010

Kong, X. J. & Zhou, C. (2015). The effect of Medilac-s combined with lactulose in the treatment of adult functional constipation. *Modern Digestion & Intervention* 20, 92-95. doi: 10.3969/j.issn.1672-2159.2015.02.004

Liang, L. N., Fan, X. Q., Yu, Z. G., Lu, S. M., Liu, L. N. & Li, C. Y. (2016). Therapeutic effects of lactulose in combination with live combined Bacillus subtilis and Enterococcus faecium enteric-coated Capsuless in elderly patients with chronic functional constipation. *World Chinese Journal of Digestology* 24, 316-321. doi: 10.11569/wcjd.v24.i2.316

Liu, H. B., fang, J. W., Xiong, J. & Yu, S. Q. (2013). Effects of Medilac-S combined with Lactulose on treating functional constipation in geriatrics. *Jiangxi Medical Journal* 48, 111-114, 120. doi: 10.3969/j.issn.1006-2238.2013.02.007

Lv, W., Li, Y. J., Xia, J. & Sun, T. (2017). Effect of live combined b.subtilis and e.faecium enteric-coated Capsuless combined with mosapride on functional constipation and analysis of clinical observation in personnel undertaking long voyage. *Translational Medicine Journal* 6, 103-105. doi: 10.3969/j.issn.2095-3097.2017.02.010

Qi, H. Y. (2018). Effects of live combined Bacillus subtilis and Bacillus subtilis enteric-coated Capsuless and lactitol on senile constipation. *Modern Diagnosis and Treatment* 29, 2367-2369. doi: 10.3969/j.issn.1001-8174.2018.15.008

Wu, X. L., He, Y. B. & Wu, Y. M. (2016). Therapeutic Efficacy of Lactulose Combined with Bacillus subtilis and Enterococcus faecium Preparation on Chronic Constipation. *Chinese Journal of Gastroenterology* 21, 90-92. doi: 10.3969/j.issn.1008-7125.2016.02.006

Zhang, S. Y. (2019). Effect of lactulose combined with live combined bacillus subtilis enteric-coated Capsuless on senile functional constipation. *Chinese Journal of Rural Medicine and Pharmacy* 26, 30-31. doi: 10.3969/j.issn.1006-5180.2019.02.018
