# Supplementary figures and images for: Efficacy and safety of live combined Bacillus subtilis and Enterococcus faecium in patients with constipation: a meta-analysis of randomized controlled trials
Source: Front Pharmacol. 2025 Oct 15;16:1688544. doi: 10.3389/fphar.2025.1688544 (PMC12569391; doi:10.3389/fphar.2025.1688544)

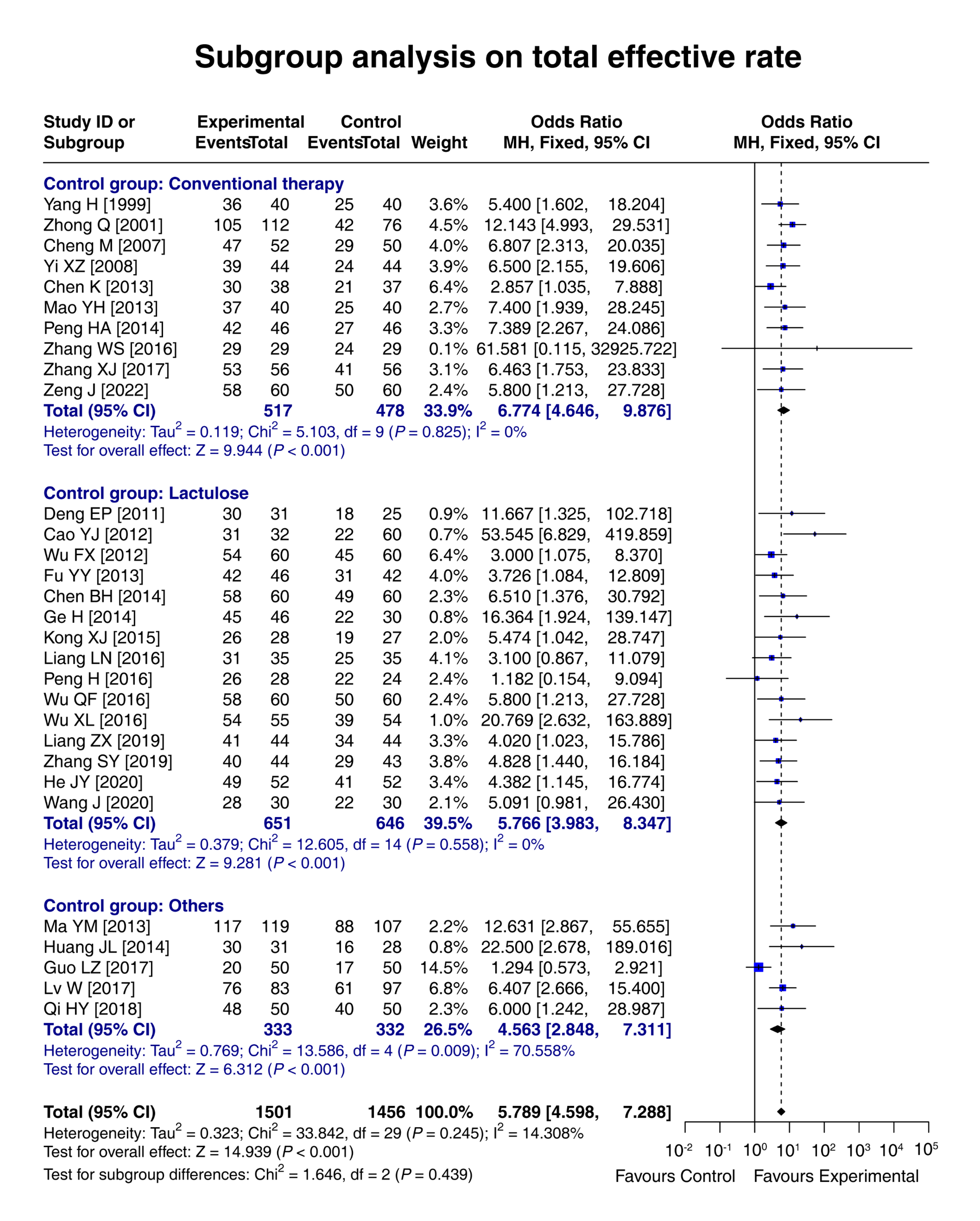

Supplement: Supplementary file 2 [file Image1.tif]
